# Exploring Heterosis in Melon (*Cucumis melo* L.)

**DOI:** 10.3390/plants9020282

**Published:** 2020-02-21

**Authors:** Marco Napolitano, Niccolò Terzaroli, Subash Kashyap, Luigi Russi, Elen Jones-Evans, Emidio Albertini

**Affiliations:** 1 Bayer, Kaiser-Wilhelm-Allee 1, 51373 Leverkusen, Germany; marco.napolitano@bayer.com (M.N.); subash.kashyap@bayer.com (S.K.); elen.jonesevans@bayer.com (E.J.-E.); 2 Dipartimento di Scienze Agrarie, Alimentari e Ambientali, Università degli Studi di Perugia, Borgo XX Giugno 74, 06121 Perugia, Italy; niccolo.terzaroli@studenti.unipg.it (N.T.); emidio.albertini@unipg.it (E.A.)

**Keywords:** breeding, diallel, epistasis, GCA, genetic distance, GGE biplot, Griffing’s method, heritability, SCA

## Abstract

Heterosis is the superiority of an F_1_ hybrid over its parents. Since this phenomenon is still unclear in melon, a half diallel experiment based on eight genetically distant breeding lines was conducted in six environments of Central Italy, assessing commercially important traits: yield, total soluble solids (TSS), and days to ripening (DTR). To estimate the additive (general combining ability; GCA) and the non-additive gene effects (specific combining ability; SCA), yield was analyzed by Griffing’s methods two and four, and the results were compared to the GGE (Genotype plus Genotype by Environment interaction) biplot methodology; TSS and earliness were evaluated only by Griffing’s method four. Overall, GCAs were significantly more relevant than SCAs for all examined traits. Least square means (LsM), mid-parent heterosis (MPH), best-parent heterosis (BPH), as well as Euclidean and Mahalanobis’ distances were calculated and compared with the genetic distance (GD). As a few correlations were found statistically significant (only for TSS), it was difficult to predict the value of a hybrid combination only by knowing the genetic distance of its parents. Despite this, heterosis was observed, indicating either the presence of epistatic effects (additive × additive interactions) and/or an underestimate of SCAs embedded within Griffing’s method. The significant Env × Entries source of variation suggests development of hybrids in specific environments. The results are discussed with a breeding perspective.

## 1. Introduction

Melon (*Cucumis melo* L.) is a diploid species (2*n* = 2*x* = 24) belonging to the *Cucurbitaceae* family; it is divided into ssp. *melo*, which includes Western melon cultivars (Cantaloupe, Galia, Honeydew, Western shippers, Piel de Sapo, and Christmas melon) and ssp. *agrestis,* including wild types from India and Japan [1]. Melon is an important crop worldwide. In 2017, the global production of cantaloupes and other melons was about 31.9 M tons on a harvested area of 1.2 M ha. Most of the production was recorded in Asia (24.1 M tons, 75.6%) with China being the leading producer (17 M tons). The Americas produced about 3.6 M tons, whereas Europe produced about 1.8 M tons [2].

Melon breeding programs pursue three main goals: (i) increase yield and earliness, (ii) improve fruit quality, and (iii) progress with disease and pest resistances. Yield and fruit quality are key traits, while earliness is crucial for early greenhouse productions. The improvement of fruit quality is a complex trait, as it includes external (size, shape, and net) and internal fruit appearance (color and taste). Fruit flavor depends on a number of factors including aromatic profile, flesh consistency, and total soluble solids content (TSS, also defined as °Brix). The latter is based on sugar concentration, and it is a reliable indicator of internal quality [3]. A crucial step in a melon breeding program is the identification of promising lines to combine generate hybrids with performances superior to the most grown varieties. In this regard, the adoption of diallel cross designs is useful in estimating the general combining ability (GCA) of parents and the specific combining ability (SCA) of hybrids, as detailed in Griffing [4]. Christie and Shattuck published an extensive review on diallel cross applications [5]. In Griffing’s analysis, the GCA is due to the additive and the additive × additive (*aa*) interactions, while the SCA is due to dominance effects and additive × dominant (*ad*) and dominant × dominant (*dd*) interactions [6].

Beside Griffing’s analysis, Yan and Hunt [7] proposed the use of GGE biplot for diallel data interpretation. Yan [8] and Yan et al. [9] were the first to suggest the use of a GGE biplot to analyze an environment-centered yield data. It was termed GGE biplot to emphasize that it displays both genotype main effect (G) and genotype × environment interaction (GE). Dehghani et al. [10] used GGE methodology for a genetic analysis of yield and related traits in a diallel scheme with seven melon populations.

One of the most powerful tools at breeder’s disposal is the assessment of the heterotic effect of hybrid combinations. Heterosis, or hybrid vigor, measures the phenotypic superiority of F_1_ hybrids over their parents, and it is used for traits such as growth rate, biomass production, and fertility. The superiority of hybrids in terms of yield, fertility, and resistance to biotic and abiotic stresses is being exploited in several crop species [11]. Although heterosis has been known and practically used for more than a century [12,13], many of its mechanisms are still largely unknown. However, in recent years, the use of new tools and approaches such as transcriptomics, proteomics, metabolomics, and epigenomics is generating better knowledge of the phenomenon [14]. Heterosis in melon was investigated, but results were strictly dependent on parents and traits considered. In “Piel de Sapo” type, for example, José et al. [15] found no heterosis for soluble solid concentration from negative to positive heterosis for fruit weight and diameter and a general positive heterosis for ovary shape, fruit length, and fruit shape.

The genetic distances inferred from molecular markers were suggested by Melchinger [16] as a promising tool for grouping germplasm sharing similar genomes and identifying heterotic patterns. In fact, most of the studies carried out in maize showed that there is a clear and positive correlation between genetic divergence of parental lines and potential heterosis, but Tomkowiak et al. [17] highlighted that the magnitude of the phenomenon could be lower than expected. Thus, while the genetic divergence is a necessary condition, at the same time, it could not be a sufficient guarantee of heterotic performances [18]. In melon, Luan et al. [19] reported that, despite differences in performance detected between parents and among F_1_ hybrids, only for branch number, a significant correlation between genetic distance and heterotic effect was evident.

Therefore, the present study was designed to assess the variation of F_1_ performances, GCA and SCA effects, and heterosis for yield per plant, TSS, and earliness in melon, traits considered to be of the highest commercial value for melon breeding programs in order to generate superior inbred lines to be used as parents. Variance components and broad- and narrow-sense heritabilities were estimated as well as the correlation of genetic distance (GD) with least square means (LsM), SCA, mid-parent heterosis (MPH), best-parent heterosis (BPH), and Euclidean and Mahalanobis’ distances in order to predict F_1_ performance.

## 2. Results

In the present study, eight inbred lines of melon (Table 1) and the 28 hybrids obtained by crossing them in a half diallel design were used to investigate heterosis in two different environments, Latina and Perugia, across three years (see Materials and Methods).

### 2.1. Yield Per Plant

The ANOVA results for yield per plant according to Griffing’s methods two and four (with genotypes as fixed and replicates as random effects) are reported in Table 2. Even if we used both methods, the differences of the genetic variances suggested to omit method two from consideration [20]. Highly significant mean squares for all sources of variation (*p* < 0.001) indicated ample differences amongst the six environments and amongst entries. Significant G × E interaction recommended looking at the variation in each environment separately.

The GCA variance was several times higher than SCA’s (GCA/SCA = 7.8), clearly indicating that additive gene actions are more important than non-additive ones. Moreover, the ANOVA for yield per plant carried out in each environment always showed significant differences at the entries and the GCA sources (Table 3), while SCA was significant only in 2015 and in Latina 2016. GCA values of PI414723 and PI161375 were the highest (Table 4), contributing with values up to 4.056 and 3.161 Kg plant^-1^, respectively. In fact, the highest ranking hybrids (LsM) were those having them as parents in combination with Ita1, Ogen, and Magyar Kincs and between themselves (Table 5). On the other hand, Vedrantais, Top Mark, and Ita1 had the lowest GCAs; the crosses Ita1 × Top Mark in Perugia 2015 and Latina 2016 ranked at the bottom of the list, but the worst performances across the three environments were recorded by Ita1 × Top Mark and Vedrantais × Ita1. Ogen × Magyar Kincs ranked almost at the bottom in both years in Latina. It is evident that parents from the same or from a close genetic cluster (Table 1) gave rise to low performing hybrids in terms of SCA as well. In fact, SCA values of crosses whose parents were PI414723 or PI161375 were most often the highest, but PI414723 × PI161375 ranked last in Perugia 2015 and midway in Latina 2015 and 2016, most probably due to similar genetic assets. Despite this, the genetic distances were not correlated with LsM and SCA effects (data not shown).

Orthogonal comparisons between hybrids and parents for yield per plant were highly significant in all environments (Table S1) and, according to Olfati et al. [21], the significant differences indicate the presence of average heterosis or MPH. The best parent heterosis for yield was positive in 19, 23, and 27 hybrids out of 28 in Latina 2015, Perugia 2015, and Latina 2016, respectively (Table 5). In particular, the maximum BPH value (94.66) was recorded by the hybrid Top Mark × PI161375 in Perugia 2015, thus confirming its highest SCA value. In general, while in Latina PI414723 was the best contributing parent in terms of heterosis, in Perugia, the most interesting lines were PI161375 and Hale’s Best Jumbo. Therefore, on the basis of LsM, it can be stated that, in Latina, PI414723 performed the best in crossing with Vedrantais, Ita1, Ogen, and Magyar Kincs, while in Perugia it was PI161375 performing the best with Ogen, Top Mark, and Hale’s Best Jumbo.

Apart from the value 0.18 found in Latina 2015 (not reliable because the GCA variance was not significant), the narrow sense heritability estimates for yield per plant (Table 6) ranged from 0.51 (Latina 2014 and Perugia 2015) to 0.77 (Perugia 2014).

These values are in agreement with those reported by Feyzan [22], Zalapa et al. [23,24], and Kalb and Davis [25]. Although traits such as yield are generally strongly polygenic, the heritability estimates from the present experiment indicate that it is possible to achieve good selection gains. At the same time, since genetic distance was not even correlated with BPH and MPH, it is difficult to predict the yield of a hybrid only by this kind of genomic tool, as reported also by Kaushik et al. [26].

**Figure 1:**
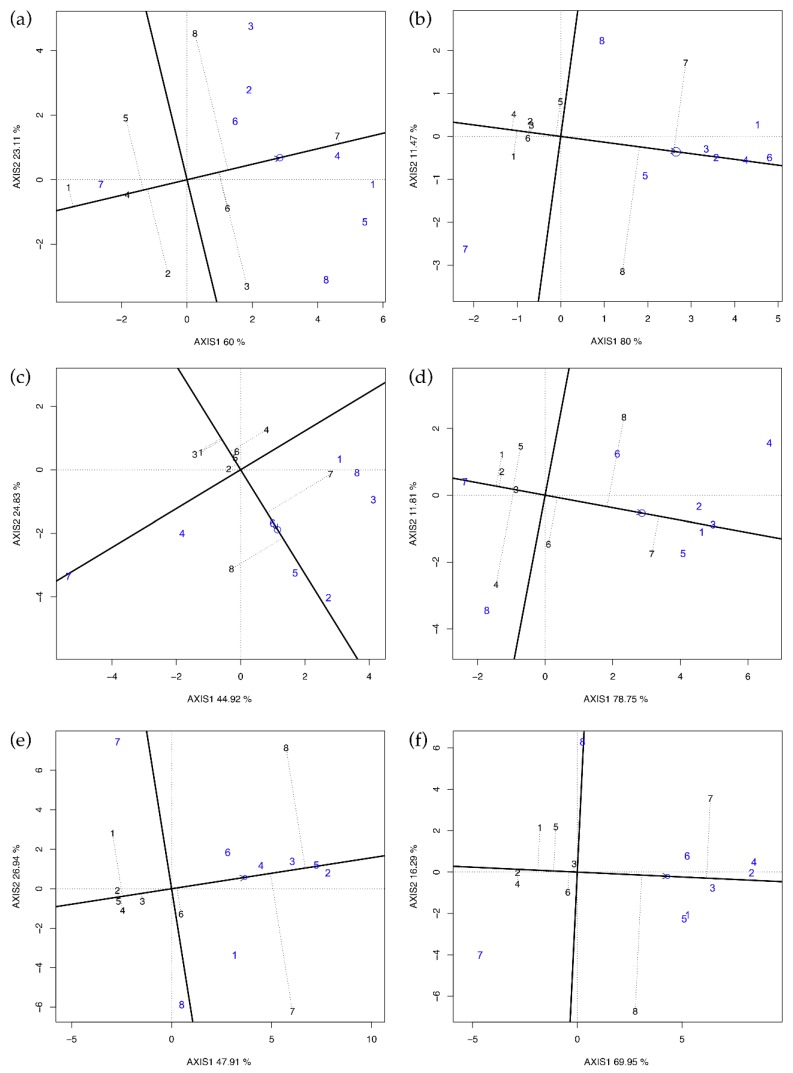
GGE biplot for yield per plant in every single environment. (**a**) Latina 2014, (**b**) Perugia 2014, (**c**) Latina 2015, (**d**) Perugia 2015, (**e**) Latina 2016, and (**f**) Perugia 2016. Parents used as entries/testers are indicated with black/blue numbers (1 = Vedrantais; 2 = Ita1; 3 = Ogen; 4 = Top Mark; 5 = Magyar Kincs; 6 = Hale’s Best Jumbo; 7 = PI414723; 8 = PI161375). Entries GCA effects are approximated by their projections on the average tester coordinates (ATC) abscissa indicated by the arrow. SCA effects are orthogonal to GCA; therefore, the projections of the entries onto ordinates of ATC must approximate their SCA effects with all the testers. If entries and testers are on the same side of the ATC abscissa, their interaction is positive, and it is negative otherwise.

GGE biplot was used to validate the results of Griffing’s method four, as it is able to display graphically and simultaneously the GCA values of all parents and their best combinations (SCA values). The method is similar to the GGE biplot used in multi-environment trials data analysis. In Latina 2015 (Figure 1c), the GCA ranking was PI161375 > PI414723 > Ita1 ≈ Magyar Kincs ≈ Hale’s Best Jumbo ≈ Top Mark > Vedrantais ≈ Ogen; this is in accordance with the Griffing’s GCA ranking reported in Table 4, except for Ogen ranking fifth rather than last. PI414723 showed the best SCA values with the testers Ogen, Vedrantais, Ita1, and PI161375 but the lowest with Top Mark and PI414723; the opposite was true for PI161375. Comparing these results with those reported in Table 4, we found again a close agreement. Confining the comments only to the results where the SCAs were significant (Latina and Perugia in 2015 and Latina in 2016, in Figure 1c–e, respectively), it is clear that Vedrantais and PI161375 were always on the same average tester coordinates (ATC) side, while Top Mark and PI414723 were on the opposite side.

**Figure 2:**
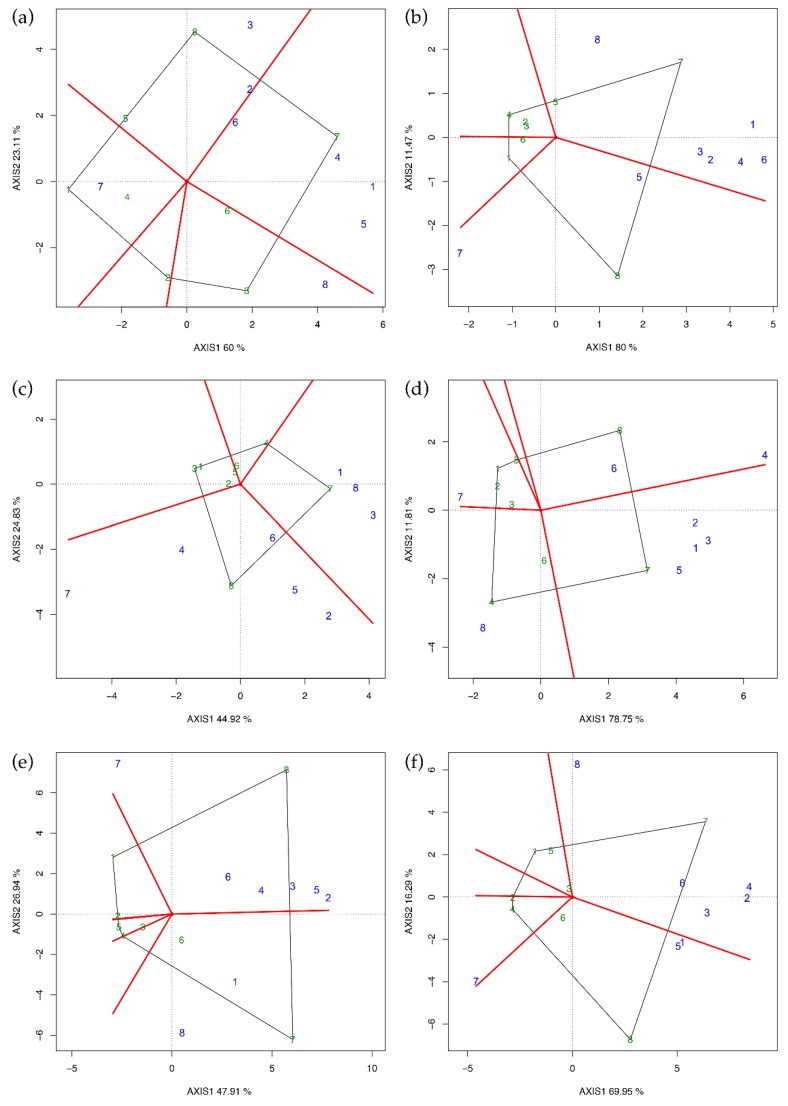
GGE biplot (polygon view) for yield per plant in every single environment. (**a**) Latina 2014, (**b**) Perugia 2014, (**c**) Latina 2015, (**d**) Perugia 2015, (**e**) Latina 2016, and (**f**) Perugia 2016. Parents used as entries/testers are indicated with green/blue numbers (1 = Vedrantais; 2 = Ita1; 3 = Ogen; 4 = Top Mark; 5 = Magyar Kincs; 6 = Hale’s Best Jumbo; 7 = PI414723; 8 = PI161375).

The polygon view (Figure 2) was obtained by joining the vertex of the entries whose coordinates were furthest from the plot origin (black lines) and dividing the polygon into sectors (red lines). It is possible to spot the best hybrid LsM by identifying the testers falling in the same sector where the entry is at the vertex. If a tester falls into the sector of its own entry, selfing is superior to crossing, and heterosis is low or nil. This was reported by Dehghani et al. [10] in a diallel scheme using Iranian landraces, but we did not find a similar pattern in any environment of our investigation because selfed parents always fell into opposite sectors.

The entries at the vertex with the largest distances from the origin are more responsive than others to the change of testers [7]. Indeed, in the case of Latina 2015 (Figure 2c), for example, GGE biplot indicates that PI414723 and PI161375 were the best mating parents, while Vedrantais and Top Mark were the poorest. Therefore, PI414723 provides the best hybrid combination with Vedrantais, Ogen, and PI161375, while PI161375 does the same with Ita1, Top Mark, Magyar Kincs, Hale’s Best Jumbo, and PI414723. Comparing Figure 2 with the results reported in Table 5, it is possible to confirm that the GGE biplot is suitable in easily spotting the best combiners and thus to validate Griffing’s results.

Concerning Perugia 2015 (Figure 2d), tester eight in sector four was predicted to be the best mating partner for Top Mark and tester four in sector eight was predicted to be the best partner for PI161375. Top Mark and PI161375 were, therefore, identified to be the best partners to one another and, according to Yan and Hunt [7], Top Mark × PI161375 must be the best of all possible combinations. For the same reason, also Vedrantais × PI414723 was another superior cross in Perugia 2015. Comparing these findings with the results reported in Table 5, we could not identify heterotic groups or patterns for yield per plant.

### 2.2. Total Soluble Solids (TSS)

Except for SCA and Env × SCA, all sources of variation for TSS in combined ANOVA (Table 2) were highly significant (*p* < 0.001), requiring a separate analysis for each environment. Similarly, yield per plant entries and GCA sources were always significant, whereas SCA was never significant, indicating for this trait only additive gene actions (Table 3). GCA values of Ita1 and Vedrantais were the highest (up to 1.712 and 1.502 °Brix, respectively), while those of Magyar Kincs and PI414723 were the lowest (Table 4). In particular, PI414723 ranged from −1.078 to −2.496 °Brix. For TSS, the GCA variances across environments were high and always significant, whereas the SCAs were too low to be significant, and thereby the estimates of narrow and broad sense heritability were identical and ranged from 0.27 in Latina 2014 to 0.49 in Perugia 2015 (Table 6).

By examining Table 4, it is evident that PI414723 was the best contributing parent for yield and, at the same time, the lowest in TSS, and the opposite was true for Vedrantais. Concerning LsM, ITA1 × Top Mark and Vedrantais × Ita1 ranked almost always at the top, followed by Vedrantais × Hale’s Best Jumbo and Vedrantais × PI161375 (Table 7). With the exception of Perugia 2014, orthogonal comparisons for TSS always showed a strong superiority (*p* < 0.001) of hybrids over parents (Table S1), indicating the presence of heterosis also for this trait. Looking at the MPH values, the positive contribution of PI161375 in increasing TSS in many crosses is evident. In fact, with the exception of Perugia 2014 (with as many as 18 negative values out of 28), Vedrantais × PI161375 ranked almost at the top in all environments, and similar behavior was shown by PI414723 × PI161375. Examining BPH values and excluding Perugia 2014 (with 23 negative values out of 28), we observed the same trend—the highest heterosis was recorded in almost all crosses with PI161375, even with PI414723, which resulted in the worst parent. Even if PI161375 did not originate hybrids with the highest LsM, it was the better parent combining with almost all other lines, and this was probably due to additive genes and additive × additive gene actions. 

Since SCA was not significant, it was not possible to correlate GD with SCA effects. However, in Perugia 2014, genetic distance showed a significant correlation with MPH (r = 0.49, *p* < 0.05) and BPH (r = 0.42, *p* < 0.05) but, as reported above, the behavior of the entries in this environment was unusual and should not be considered reliable. However, GD showed significant correlations with LsM in Latina 2015 (r = 0.46, *p* < 0.05), Perugia 2015 (r = 0.57, *p* < 0.05), and Latina 2016 (r = 0.50, *p* < 0.05), thus the genetic relationship between parents could be useful to be known in advance although insufficient to predict the TSS of a given cross.

### 2.3. Earliness

Earliness is the target of many breeding programs. It was assessed in number of days from transplant to ripening (DTR) considering only the first five fruits per plot (i.e., the first wave of fruit setting with the highest commercial importance). Low DTR values of GCA, SCA, MPH, and BPH indicate earliness of parents and hybrids.

Except for Env × SCA, the combined ANOVA sources for earliness were all highly significant (*p* < 0.001, Table 2). Looking at the ANOVAs in individual environments, SCA source was always significant except for Perugia in 2015, while entries and GCA were highly significant in all environments (Table 3). Feyzian et al. [22] reported that it is SCA that significantly affects the differences in maturity, while our results, with the exception of Latina 2016, indicate a greater importance of additive gene actions in all environments, with GCA/SCA ratio ranging from 2.23 to 7.52 (Table 6).

PI414723 always had the highest GCA, conferring to the hybrids at least three days of earliness, followed by Magyar Kincs in Perugia and by Vedrantais in Latina (Table 4). In fact, the earliest ripening hybrids were Magyar Kincs × PI414723, Vedrantais × PI414723 and PI414723 × PI161375 (Table 8). Conversely, Ita1 was the line mostly contributing to lateness; Ita1 × Top Mark, Ita1 × PI161375, Ita1 × Ogen and Ita1 × Magyar Kincs were amongst the latest ripening hybrids. Interestingly, the crosses Vedrantais × Hale’s Best Jumbo, Vedrantais × Magyar Kincs and Vedrantais × PI161375 were the earliest in Latina but amongst the latest in Perugia. These differences were mostly due to the contrasting number of days to ripening shown by the parents in the two locations, with a difference in DTR for the same parent ranging from six to 13 days (data not shown). Concerning SCA rankings, there was a trend across the five environments, with some crosses often at the top (i.e., Top Mark × PI161375, Ita1 × Hale’s Best Jumbo, Vedrantais × Ita1 and Ita1 × PI414723) and some others consistently at the bottom (Ita1 × PI161375 and Vedrantais × Top Mark). Above all, Ita1 × PI161375 was always characterized by high SCA and late ripening values.

Interestingly, orthogonal comparisons between parents and hybrids for DTR were highly significant (*p* < 0.001) in Perugia in all years and significant in Latina (*p* < 0.05) only in 2016 (Table S1). In all cases, these differences were negatives, indicating that the pools of hybrids were ripening earlier by a few days compared to the parents, therefore indicating the effect of heterosis. In fact, BPH values showed an opposite trend between the two sites; in Perugia 2014 and 2016, as many as 18 and 17 out of 28 hybrids, respectively, showed negative values, while in Latina, we found only four, one, and five out of 28 hybrids showing heterosis for earliness (Table 8). Moreover, in Perugia, the crosses with PI414723 as a parent, i.e., Hale’s Best Jumbo × PI414723, Top Mark × PI414723, Vedrantais × PI414723 and Ita1 × PI414723, showed the lowest BPHs, while in Latina, their BPHs were positive. 

Narrow sense heritability for earliness (Table 6) ranged from 0.41 in Perugia 2016 to 0.82 in Latina 2015. Examining all values together, the narrow sense heritability was always higher in Latina than in Perugia, indicating that, in the case of selection for earliness, this must be conducted separately in each location, and Latina seems to be more suitable than Perugia, as resulted from the magnitude of their respective error variances (σ^2^_E_).

Similar to yield per plant, for earliness, no significant correlations were found between GD on one side and LsM, SCA effects, BPH, and MPH values on the other. Even using all traits together in a multivariate dimension (Mahalanobis’ and Euclidean distances), it was not possible to find a correlation with GD.

## 3. Discussion

The main goals of the present paper were to assess melon hybrids performances for a number of important commercial traits, to estimate gene effects and heterosis, and to detect any correlation between them and the genetic distance of their parents.

Additive gene effects showed themselves to be the most important genetic component for all traits examined (yield per plant, TSS, and earliness); as a result, the narrow sense heritabilities were found rather high, indicating the possibility of achieving good selection gains. Moreover, our data indicated a central role of epistasis. In fact, a significant heterosis was recorded for yield and TSS, and this can be explained entirely by *aa* epistatic effects included in GCA [27]. Additionally, since SCA was not significant, dominant and *ad* and *dd* epistatic gene effects are likely not to influence heterosis. The lack of correlations between heterosis and GD confirms that the latter is not able to predict the former, since GD is unable to take epistatic interactions into account.

Several authors [25,28,29,30,31] reported that yield per plant in melon is mostly based on the additive effect of genes. Our ratio between GCA and SCA equal to 7.8 confirmed this. Moreover, comparing Figure 2 with Table 5, it is possible to confirm that the GGE biplot is suitable in easily spotting the best partners and thus to validate Griffing’s results. The same trend was observed for single fruit weight, dimensions (length and diameter), and fruit shape (Table S2). Although yield is a strongly polygenic trait [23], the narrow sense heritability estimates were indeed remarkable, ranging from 0.51 to 0.77. Best-parent heterosis was consistent and up to 94.66; this phenomenon can be explained to a small extent by the dominance effects comprised in SCA but more so by aa interactions. Genetic distances were not correlated with LsM, SCA effects, or BPH and MPH, thus it can be difficult to predict the yield of a hybrid only with this kind of genomic tools [26].

When SCA source is not significant, as is the case of TSS, Baker [32] suggests that the value of the hybrids could be predicted by the GCAs of the parents. By comparing the GCA values of parents (Table 4) and the means of all entries in each environment (Table 7), the suggestion of Baker is of practical relevance at least for the top and the bottom ranking hybrids. These results are in accordance with Akrami and Arzani [33]. Despite the absence of dominance effects, MPH and BPH were still relevant, probably due to *aa* epistatic effects, as for yield per plant. Only for TSS did we find significant correlations of GD with LsM in three out of five environments (r = 0.46, 0.50, 0.57, *p* < 0.05), thus an a priori knowledge of the genetic background of the possible parents could be useful to create pools to maximize the sugar content of the hybrids.

Feyzian et al. [22] suggested the importance of non-additive gene actions, since their GCA/SCA ratio for earliness was 0.3. Conversely, our GCA/SCA ranged from 2.23 to 7.52, clearly indicating that GCA effects surpass SCA. Indeed, narrow sense heritability ranged from 0.41 to 0.82, both values higher than the 0.23 reported by Feyzian et al. [22] but consistent with the 0.61 reported by Kalb and Davis [25]. The magnitude of heterosis, ranging from ±10%, and its variability across trials suggest developing superior parental lines specifically adapted to each environment and to consider earliness as an additive trait. For earliness, such as for yield per plant, the predictability of hybrid performances is negligible, as the correlations between GD with SCA, heterosis, or LsM are not significant.

Moreover, Kalb and Davis [25] hypothesized that the geographical and, hence, the genetic distance among American melon cultivars were positively correlated with good performances. Napolitano [34], in attempts to correlate GD with heterosis, suggested that molecular markers could be used for parent selection when pedigree data are not available. However, even using all traits together in a multivariate dimension (Mahalanobis’ and Euclidean distances), as indicated by several authors [35,36,37,38], it was not possible to find a correlation with GD. Most likely, the markers used primarily assessed neutral genome regions not involved in controlling the traits of our study. However, this method can be a viable route when there are no associated markers with the desired characters or for pre-breeding studies. It can also be useful whenever SCA can be omitted from the model because it is not significant (i.e., TSS), but it clearly shows the difficulties of predicting hybrid performances and heterosis even starting from genetically characterized parents.

## 4. Conclusions

In the present study, the significant Env × Entries source of variation clearly indicates that hybrids are expected to be more successful when developed for a specific environment than across environments; lines will be best adapted to specific conditions, as reported for important traits such as yield per plant, TSS, and earliness. Moreover, since the additive effects included in GCA were more consistent than the non-additive ones (SCA), breeding for all of these traits needs to be addressed to increase line performances per se. In fact, mid-parent heterosis is expected to decrease proportionally due to accumulation of favorable dominant alleles at individual quantitative trait loci (QTL), but heterosis can be maintained by exploiting the additive × additive effects present in the parent combinations [39]. As a consequence of this, reciprocal recurrent selection [40] of the best lines from different genetic pools is expected to generate superior inbred lines to be used as parents, summing up general and specific combining abilities and heterosis as well.

## 5. Materials and Methods

### 5.1. Plant Material and Genetic Distance

In the present study, eight inbred lines of melon (*Cucumis melo* L.) were analyzed. According to the classification of Renner and Schaefer [41], six of the selected lines belong to *C. melo* spp. *melo* (Ita1, Vedrantais, Top Mark, Hale’s Best Jumbo, Ogen, Magyar Kincs) and two to *C. melo* spp. *agrestis* (PI414723, PI161375) (Table 1, Figure S1). The eight parental lines were crossed according to a half diallel design covering all possible combinations (28 F_1_ hybrids) without reciprocals.

The genetic distance matrix among parental lines (Table S3) was obtained in a preliminary molecular fingerprinting experiment [34] using 7684 single nucleotide polymorphisms (SNPs). The Unweighted Pair Group Method with Arithmetic mean (UPGMA) clustering and Principal Coordinate Analysis (PCA) provided a fairly good division of parental lines in four distinct genetic clusters (Table 1).

### 5.2. Field Trials and Data Recording

The morphological evaluation and the comparison of the productivity of the eight parental lines and their 28 F_1_ hybrids were carried out in the summers of 2014, 2015, and 2016 in two different locations: Bayer Crop Science R&D Site in Latina, Italy (41°27′42.8′′ N, 12°45′18.4′′ E, alt. 13 m) and Papiano, the experimental farm of the University of Perugia, Italy (42°57′21.9′′ N, 12°22′32.7′′ E, alt. 165 m). The two locations were representative of the melon production areas in Italy; during the growing season (May–August), Latina was characterized by average minimum, mean, and maximum temperatures of 17.1, 25.2, and 28.6 °C, respectively, vs. 17.2, 21.9, and 25.7 °C of Perugia. The mean total rainfalls during the same period were 126.6 and 218.2 mm in Latina and Perugia, respectively. The commercial hybrid (SV9424ML, Seminis) was used as a control. Four young plants per entry, previously grown in jiffy pots, were transplanted in the fields in plots of 0.8 m long and 2.5 m wide, arranged in a randomized complete block design with three replicates.

The cultivation protocol ensured optimal growing conditions throughout the season, applying the recommended rates of fertilizer and irrigation. Particular attention was given to the control of pests and diseases, as the level of genetic resistance among the entries was variable. Data were collected daily on a single-fruit basis for the following traits: (1) earliness (DTR, days to ripening as days between transplant and maturity only for the first five fruits per plot; fruits were considered ripe after inspecting the abscission layer, the “ring” between peduncle and fruit, and the change of fruit skin color); (2) fruit weight (FW, in kilograms); (3) fruit length (FL, in centimeters); (4) maximum fruit diameter (FD, in centimeters); (5) fruit shape (FS, as the ratio FL/FD); and (6) total soluble solids (TSS, in °Brix, using a refractometer to analyze a drop of juice extracted from the equatorial region of the mesocarp).

### 5.3. Statistical Analysis

Prior to any statistical analysis, data were tested for skewness, kurtosis, and subjected to a Q-Q (quantile-quantile) plot for normality by R statistical software Ver. 3.5.3 [42]. Combined and single site analyses of variance were performed for each variable by the software Analysis of Genetic Design using R (AGD-R) [43]. Only for yield per plant, GCAs and SCAs were calculated by Griffing’s [4] method two and method four and using Model B with Genotypes as fixed effects and replicates as random in both cases. Broad and narrow sense heritability were estimated by Model 2 (all effects random). Griffing’s method four Model B (excluding parents, as recommended by Yao et al. [20]) was used for earliness and fruit traits such as weight, length, diameter, shape, and TSS. Orthogonal comparisons between hybrids and parents for yield per plant, TSS, and DTR were carried out with “emmeans” R package [44]. MPH and BPH of each trait were calculated as follows:

(1) $$MPH=\frac{F_{1}-\left(\left(P_{1}+P_{2}\right)/2\right)}{\left(\left(P_{1}+P_{2}\right)/2\right)}\times 100$$

(2) $$BPH=\frac{F_{1}-P_{h}}{P_{h}}\times 100,$$

where *F*_1_ is the mean of the hybrid with parents *P*_1_ and *P*_2_, and *P*_h_ is the mean of the better parent between them. Standard errors for testing the significance of *MPH* and *BPH* were calculated as follows:

(3) $$SE_{MPH}=\sqrt{3Me/2r}$$

(4) $$SE_{BPH}=\sqrt{2Me/r}$$

where *Me* is the error mean square from the ANOVA table, and *r* is the number of replications [45].

The diallel data of yield per plant were also analyzed by GGE biplot by averaging each cross over replications and including selfed parents. The statistical significance of “Environments” and of “G × E” source of variation in the combined ANOVA suggested to carry out the GGE biplot for each of the six environments. Each genotype was considered both an entry and a tester. Means of each tester were calculated, and an adjusted matrix was obtained by subtracting the tester mean from each cell. Details of the model are described in Yan and Hunt [7]. GGE analysis was carried out by the R package GGEBiplotGUI [46].

Camussi et al. [35] and Lefebvre et al. [47] suggested the use of distances obtained by multivariate analysis of morphological data, including that for a diallelic scheme. The continuous distribution of polygenically controlled traits may be particularly useful in intergroup classification in terms of geometrical distances, such as the D^2^ of Mahalanobis [48]. The Euclidean distances were calculated in a multivariate space using all phenotypic traits described above, while the Mahalanobis’ distances were calculated through a canonical analysis. Both distances were obtained for each of the six environments. The genetic distance matrix, previously assessed in another study [34], was then compared with LsM, SCA, BPH, MPH, and Euclidean and Mahalanobis’ distance matrices by the Mantel test [49] using the R package “biotools” [50] with 10,000 permutations.

## Figures and Tables

**Table 1:** Parental lines of melon used in the diallel, classified according to Pitrat (2008).

N	Accession Name	Subspecies	Botanical Group	Genetic Cluster
1	Vedrantais	*melo*	*cantalupensis*	C1
2	Ita1	*melo*	*reticulatus*	C1
3	Ogen	*melo*	*cantalupensis*	C3
4	Top Mark	*melo*	*reticulatus*	C2
5	Magyar Kincs	*melo*	*reticulatus*	C3
6	Hale’s Best Jumbo	*melo*	*reticulatus*	C2
7	PI414723	*agrestis*	*momordica*	C4
8	PI161375	*agrestis*	*chinensis*	C4

**Table 2:** Combined ANOVA for yield per plant (Kg), total soluble solids (TSS, °Brix), and earliness (DTR) across the six environments used in the experiment.

	Yield G2 ^†^			Yield G4			TSS			Earliness		
	df	MS		df	MS		df	MS		df	MS	
Environments	5	329.46	***	5	763.43	***	4	6504.65	***	5	3696.62	***
Entries	35	28.03	***	27	62.32	***	27	634.81	***	27	712.42	***
GCA	7	82.95	***	7	216.06	***	7	86.73	***	7	456.55	***
SCA	28	14.31	***	20	8.51	***	20	3.64		20	32.16	***
Env × Entries	175	3.29	***	135	5.12	***	108	54.38	***	135	44.62	***
Env × GCA	35	6.55	***	35	7.84	***	28	5.670	*	35	18.93	***
Env × SCA	140	2.48	***	100	4.17	**	80	0.66		100	5.50	
Error	420	1.35		324	2.86		11,169	3.89		2335	4.61	
GCA Variance (σ^2^_g_)		0.674	**		1.888	***		0.867	***		3.805	***
SCA Variance (σ^2^_s_)		2.559	***		0.242	*		0.199	***		1.481	***
GCA × Env Variance		0.124	***		0.204	***		0.280	***		0.746	***
SCA × Env Variance		0.648	***		0.436	***		0	***		0.299	***
Error Variance (σ^2^_E_)		2.651			2.860			3.889			4.607	
Additive Variance (σ^2^_A_)		1.349			3.775			1.734			7.611	
Dominance Variance (σ^2^_D_)		2.559			0.242			0.199			1.481	
Phenotypic Variance (σ^2^_P_)		6.558			6.877			5.821			13.698	
GCA–SCA ratio (σ^2^_g_/σ^2^_s_)		0.263			7.816			4.360			2.570	
Narrow sense Heritability (h^2^_N_)		0.206			0.549			0.298			0.556	
Broad sense Heritability (h^2^_B_)		0.596			0.584			0.332			0.664	

*, **, *** Significant at 0.05, 0.01, and 0.001 probability level, respectively. ^†^ G2 and G4 are referred to Griffing’s methods two and four, respectively. GCA: general combining activity; SCA: specific combining activity.

**Table 3:** ANOVA results for yield per plant (Kg), total soluble solids (TSS, °Brix), and earliness (DTR) in each environment.

		Yield			TSS			Earliness		
		df	MS		df	MS		df	MS	
Latina 2014	Entries	27	10.93	***	27	128.38	***	27	77.08	***
	GCA	7	32.12	***	7	12.53	***	7	49.42	***
	SCA	20	3.52		20	1.08		20	3.49	***
	Error	54	2.83		2730	3.44		385	1.17	
Perugia 2014	Entries	27	8.2	***	27	70.66	***	27	139.21	***
	GCA	7	28.11	***	7	14.52	***	7	77.24	***
	SCA	20	1.23		20	1.11		20	10.55	*
	Error	54	0.72		1438	3.08		390	5.56	
Latina 2015	Entries	27	9.98	**	27	171.44	***	27	162.67	***
	GCA	7	17.6	***	7	20.47	***	7	111.41	***
	SCA	20	7.32	*	20	1.42		20	4.93	***
	Error	54	4.04		2415	2.98		390	1.32	
Perugia 2015	Entries	27	12.04	***	27	179.98	***	27	143.21	***
	GCA	7	32.5	***	7	31.61	***	7	86.72	***
	SCA	20	4.87	**	20	0.92		20	8.32	
	Error	54	2.01		1706	3.51		390	6.58	
Latina 2016	Entries	27	23.03	***	27	291.74	***	27	144.14	***
	GCA	7	65.67	***	7	30.37	***	7	98.26	***
	SCA	20	8.11	*	20	1.76		20	4.58	*
	Error	54	4.41		2880	5.71		390	2.39	
Perugia 2016	Entries	27	23.73	***				27	269.13	***
	GCA	7	79.26	***				7	128.15	***
	SCA	20	4.3					20	27.81	***
	Error	54	3.04					390	10.55	

*, **, *** Significant at 0.05, 0.01, and 0.001 probability level, respectively.

**Table 4:** GCAs and relative ranking (italics) for yield per plant (Kg), total soluble solids (TSS, °Brix), and earliness (DTR) of the eight parents used in the diallel cross evaluated in six environments (LT = Latina, PG = Perugia).

		LT 2014		PG 2014		LT 2015		PG 2015		LT 2016		PG 2016	
	Parent ^†^	GCA	Rank	GCA	Rank	GCA	Rank	GCA	Rank	GCA	Rank	GCA	Rank
Yield	1	−1.876	8	−0.757	6	−0.971	8	−1.247	7	−2.355	8	−1.811	8
	2	−1.016	6	−0.729	5	−0.084	3	−1.289	8	−0.689	6	−1.685	7
	3	1.116	2	−0.767	7	−0.537	5	−0.363	4	−0.053	3	0.718	3
	4	−1.052	7	−0.851	8	−0.798	7	−0.455	5	−1.743	7	−1.559	6
	5	−0.528	5	−0.533	4	−0.105	4	−0.588	6	−0.422	5	−1.115	5
	6	0.389	4	−0.284	3	−0.551	6	−0.071	3	−0.381	4	−0.544	4
	7	2.098	1	2.440	1	1.437	2	2.592	1	2.482	2	4.056	1
	8	0.869	3	1.481	2	1.609	1	1.420	2	3.161	1	1.941	2
TSS	1	0.293	3	0.316	4	1.128	1	1.502	2	1.408	2	NA ^‡^	
	2	0.575	2	1.301	1	0.994	2	1.712	1	1.473	1	NA	
	3	0.206	4	−0.022	5	−0.312	6	0.204	3	0.355	4	NA	
	4	0.055	5	0.760	2	−0.007	5	0.059	5	−0.188	6	NA	
	5	−0.804	7	−0.913	7	−0.685	7	−0.813	7	−1.371	7	NA	
	6	−0.701	6	0.535	3	0.358	4	0.195	4	−0.124	5	NA	
	7	−1.078	8	−1.307	8	−2.138	8	−2.496	8	−2.259	8	NA	
	8	1.454	1	−0.671	6	0.662	3	−0.362	6	0.706	3	NA	
Earliness	1	−1.026	2	0.542	6	−0.458	3	−0.947	3	−0.264	2	1.925	7
	2	2.501	8	1.608	7	3.353	8	1.864	7	2.847	8	−0.297	3
	3	0.729	6	0.408	5	1.208	7	0.086	4	1.347	7	0.292	4
	4	1.140	7	2.308	8	0.819	6	2.064	8	1.036	6	3.725	8
	5	0.518	5	−0.481	2	0.697	5	−1.203	2	−0.186	3	−2.431	2
	6	−0.260	4	0.197	4	0.642	4	1.242	5	0.292	5	1.125	6
	7	−3.076	1	−4.625	1	−5.281	1	−4.492	1	−5.197	1	−4.953	1
	8	−0.526	3	0.042	3	−0.981	2	1.386	6	0.125	4	0.614	5

^†^ The names of the parents are: 1 = Vedrantais; 2 = Ita1; 3 = Ogen; 4 = Top Mark; 5 = Magyar Kincs; 6 = Hale’s Best Jumbo; 7 = PI414723; 8 = PI161375. ^‡^ NA, not available.

**Table 5:** Least square means (LsM) of yield per plant (Kg), specific combining ability (SCA), and its ranking (in italics), mid-parent heterosis (MPH), and best-parent heterosis (BPH) of the 28 crosses in environments with a significant ANOVA SCA mean square.

	Latina 2015					Perugia 2015					Latina 2016				
Cross ^†^	LsM	SCA	Rank	MPH	BPH	LsM	SCA	Rank	MPH	BPH	LsM	SCA	Rank	MPH	BPH
1 × 2	8.08	−1.372	24	3.46	−5.39	4.56	0.033	15	−7.6	−12.14	10.78	0.21	13	21.12	9.33
1 × 3	10.29	1.287	5	50.44	45.34	5.07	−0.382	17	8.33	8.33	11.84	0.633	9	32.36	18.99
1 × 4	9.48	0.744	11	14.05	1.07	4.65	−0.714	22	3.32	−0.64	10.58	1.065	6	19.95	13.16
1 × 5	9.42	−0.008	16	27.47	22.34	4.44	−0.785	23	−5.43	−5.73	11.13	0.3	12	28.89	19.29
1 × 6	9.39	0.405	13	8.74	−7.85	6.35	0.609	8	36.71	35.68	11.05	0.175	14	19.65	4.94
1 × 7	12.44	1.465	3	67.77	60.52	9.55	1.145	2	58.9	30.11	14.88	1.143	5	88.71	87.41
1 × 8	8.62	−2.519	27	−6.05	−23.51	7.33	0.095	14	39.75	26.16	10.89	−3.526	28	6.04	−13.57
2 × 3	9.45	−0.438	18	24.83	10.66	4.46	−0.951	25	−9.63	−14.07	13.35	0.483	10	34.78	35.4
2 × 4	8.56	−1.065	23	−4.46	−8.74	4.04	−1.281	27	−14.68	−22.16	10	−1.183	23	4.11	1.42
2 × 5	11.27	0.957	9	38.79	31.97	5.51	0.325	12	11.31	6.17	10.91	−1.59	25	13.71	10.65
2 × 6	9.34	−0.526	19	−0.27	−8.34	6.37	0.671	7	30	22.74	11.27	−1.274	24	10.54	7.03
2 × 7	12.91	1.054	8	58.50	51.17	9.45	1.081	3	50.84	28.75	17.17	1.765	2	94.12	74.14
2 × 8	13.42	1.391	4	35.49	19.08	7.31	0.122	13	32.91	25.82	17.67	1.589	3	57.35	40.24
3 × 4	9.56	0.389	14	19.65	1.92	5.86	−0.387	18	30.8	25.21	12.6	0.781	8	30.57	26.63
3 × 5	8.28	−1.586	25	15.8	7.53	6.12	0.015	16	30.35	29.94	10.05	−3.085	27	4.25	1.01
3 × 6	9.34	−0.08	17	11.26	−8.34	7.07	0.444	11	52.21	51.07	13.5	0.32	11	31.84	28.21
3 × 7	13.48	2.078	1	87.87	73.94	9.97	0.684	6	65.89	35.83	16.11	0.071	17	81.21	61.91
3 × 8	9.93	−1.65	26	11.14	−11.89	8.7	0.578	9	65.87	49.74	17.52	0.798	7	55.39	39.05
4 × 5	10.71	1.108	6	25.41	14.18	5.55	−0.473	19	23.47	17.83	11.46	0.014	19	22.7	22.57
4 × 6	10.73	1.578	2	9.66	5.3	5.61	−0.924	24	26.21	21.69	11.32	−0.172	20	13.88	7.5
4 × 7	7.56	−3.581	28	−11.73	−19.4	9.69	0.492	10	66.78	32.02	13.72	−0.635	22	59.72	46.74
4 × 8	12.14	0.828	10	17.58	7.72	11.31	3.286	1	124.18	94.66	15.16	0.13	15	38.13	20.32
5 × 6	9.01	−0.836	21	0.73	−11.58	7.19	0.788	4	54.29	52.65	15.91	3.097	1	60.22	51.09
5 × 7	11.12	−0.718	20	43.95	43.48	9.77	0.708	5	62.16	33.11	15.75	0.081	16	83.57	68.81
5 × 8	13.09	1.083	7	38.01	16.15	7.32	−0.577	21	39.16	25.99	17.54	1.183	4	59.96	39.21
6 × 7	10.54	−0.853	22	17.5	3.43	8.49	−1.097	26	42.09	15.67	13.52	−2.198	26	47.28	28.4
6 × 8	11.88	0.313	15	10.72	5.41	7.92	−0.491	20	52.02	36.32	16.44	0.052	18	42.15	30.48
7 × 8	14.11	0.555	12	48.37	25.2	8.06	−3.013	28	22.59	9.81	19.03	−0.226	21	86.29	51.03
S.E.	1.12	0.981		1.421	1.641	0.768	0.692		1.002	1.158	1.26	1.024		1.48	1.715

^†^ The names of the parents are: 1 = Vedrantais; 2 = Ita1; 3 = Ogen; 4 = Top Mark; 5 = Magyar Kincs; 6 = Hale’s Best Jumbo; 7 = PI414723; 8 = PI161375.

**Table 6:** Variance components of yield per plant (Kg), total soluble solids (TTS, °Brix), and earliness (DTR) in each of the six environments.

		LT 2014	PG 2014	LT 2015	PG 2015	LT 2016	PG 2016
Yield	GCA Variance (σ^2^_g_)	1.59 ***	1.49 ***	0.57	1.54 ***	3.20 ***	4.16 ***
	SCA Variance (σ^2^_s_)	0.23	0.17	1.09 *	0.95 **	1.24 *	0.42
	Error Variance (σ^2^_E_)	2.83	0.72	4.04	2.01	4.41	3.04
	Additive Variance (σ^2^_A_)	3.18	2.99	1.14	3.07	6.40	8.33
	Dominance Variance (σ^2^_D_)	0.23	0.17	1.09	0.95	1.24	0.42
	Phenotypic Variance (σ^2^_P_)	6.24	3.88	6.28	6.04	12.04	11.79
	GCA–SCA ratio (σ^2^_g_/σ^2^_s_)	6.98	8.80	0.52	1.61	2.59	9.94
	Narrow sense Heritability (h^2^_N_)	0.51	0.77	0.18	0.51	0.53	0.71
	Broad sense Heritability(h^2^_B_)	0.55	0.81	0.36	0.67	0.63	0.74
TSS	GCA Variance (σ^2^_g_)	0.64 ***	0.75 ***	1.06 ***	1.70 ***	1.59 ***	NA ^‡^
	SCA Variance (σ^2^_s_)	0	0	0	0	0	NA
	Error Variance (σ^2^_E_)	3.44	3.08	2.98	3.51	5.71	NA
	Additive Variance (σ^2^_A_)	1.27	1.49	2.12	3.41	3.18	NA
	Dominance Variance (σ^2^_D_)	0.00	0.00	0.00	0.00	0.00	NA
	Phenotypic Variance (σ^2^_P_)	4.71	4.57	5.09	6.92	8.89	NA
	GCA–SCA ratio (σ^2^_g_/σ^2^_s_)	-	-	-	-	-	NA
	Narrow sense Heritability (h^2^_N_)	0.27	0.33	0.42	0.49	0.36	NA
	Broad sense Heritability(h^2^_B_)	0.27	0.33	0.42	0.49	0.36	NA
Earliness	GCA Variance (σ^2^_g_)	2.55 ***	3.70 ***	5.92 ***	4.36 ***	5.20 ***	5.57 **
	SCA Variance (σ^2^_s_)	0.77 ***	1.66 *	1.2 ***	0.58	0.73 *	5.75 ***
	Error Variance (σ^2^_E_)	1.17	5.56	1.32	6.58	2.39	10.55
	Additive Variance (σ^2^_A_)	5.10	7.41	11.83	8.71	10.41	11.15
	Dominance Variance (σ^2^_D_)	0.77	1.66	1.20	0.58	0.73	5.75
	Phenotypic Variance (σ^2^_P_)	7.05	14.64	14.36	15.87	13.53	27.46
	GCA–SCA ratio (σ^2^_g_/σ^2^_s_)	3.30	2.23	4.93	7.52	7.14	0.97
	Narrow sense Heritability (h^2^_N_)	0.72	0.51	0.82	0.55	0.77	0.41
	Broad sense Heritability(h^2^_B_)	0.83	0.62	0.91	0.59	0.82	0.62

*, **, *** Significant at 0.05, 0.01, and 0.001 probability level, respectively. ^‡^ NA, not available.

**Table 7:** Least square means (LsM), SCA, MPH, and BPH for total soluble solids (TSS) of the 28 crosses in environments with a significant ANOVA SCA mean square.

	Latina 2014			Perugia 2014			Latina 2015			Perugia 2015			Latina 2016		
Cross ^†^	LS Means	MPH	BPH	LS Means	MPH	BPH	LS Means	MPH	BPH	LS Means	MPH	BPH	LS Means	MPH	BPH
**1 × 2**	9.28	11.20	7.66	7.48	8.01	−5.91	12.45	11.41	2.13	13.30	19.66	13.77	12.73	21.12	12.06
**1 × 3**	8.88	5.15	3.02	5.72	−8.26	−12.94	11.00	7.26	6.28	12.60	14.96	10.72	12.10	22.47	19.80
**1 × 4**	9.05	9.05	4.99	7.60	15.50	2.70	11.05	15.58	8.76	11.51	7.98	5.89	10.79	16.25	11.70
**1 × 5**	7.62	1.33	−11.60	5.07	−8.15	−14.07	11.12	15.47	9.45	11.16	21.77	5.88	11.17	31.72	15.63
**1 × 6**	8.02	5.04	−6.96	7.83	19.63	8.90	12.75	27.25	25.49	12.82	20.21	18.81	11.29	19.28	16.87
**1 × 7**	7.52	4.37	−12.76	4.78	−5.44	−18.98	8.98	11.55	−11.61	8.89	8.81	−15.65	8.89	19.49	−7.97
**1 × 8**	10.49	29.27	21.69	6.27	6.00	5.73	13.33	50.28	31.20	11.41	26.85	8.25	12.56	59.90	30.02
**2 × 3**	7.93	−2.94	−4.11	7.47	2.89	−6.04	10.80	−4.17	−11.40	12.26	6.29	4.88	12.50	16.50	10.04
**2 × 4**	9.65	19.95	19.58	8.97	16.87	12.83	12.51	19.09	2.63	12.81	13.56	9.58	12.95	26.46	14.00
**2 × 5**	8.32	14.84	3.10	5.97	−8.79	−24.91	10.28	−3.43	−15.67	10.81	10.99	−7.53	9.68	3.75	−14.79
**2 × 6**	8.76	19.02	8.55	8.41	11.10	5.79	12.42	12.55	1.89	12.32	9.61	5.39	11.57	12.17	1.85
**2 × 7**	7.91	14.14	−1.98	5.96	−1.97	−25.03	9.08	0.17	−25.51	9.17	4.86	−21.56	8.35	0.72	−26.50
**2 × 8**	10.62	35.46	31.60	6.71	−3.31	−15.60	12.66	28.07	3.86	12.08	26.23	3.34	12.09	38.89	6.43
**3 × 4**	9.58	17.62	15.84	7.23	3.51	−2.30	10.64	11.01	2.80	10.39	−6.61	−8.70	10.35	7.70	2.48
**3 × 5**	8.24	12.19	−0.36	5.01	−14.43	−23.74	10.73	10.33	3.67	9.64	0.57	−15.29	8.75	0.57	−13.37
**3 × 6**	7.71	3.35	−6.77	6.95	1.02	−3.34	10.44	3.21	0.87	11.29	1.85	−0.79	10.22	5.52	1.19
**3 × 7**	7.17	1.99	−13.30	4.91	−8.91	−25.27	8.15	0.06	−21.26	7.15	−16.76	−37.17	5.58	−27.15	−44.75
**3 × 8**	10.42	31.23	26.00	5.55	−11.20	−15.53	10.21	13.89	−1.35	10.53	11.84	−7.47	11.28	39.69	11.68
**4 × 5**	7.51	4.02	−6.36	6.36	1.44	−14.05	9.87	10.16	8.46	9.64	3.32	−11.32	7.57	−7.80	−17.00
**4 × 6**	7.00	−4.57	−12.72	7.02	−3.77	−5.14	10.02	7.17	1.42	10.33	−4.62	−4.97	9.61	4.51	3.67
**4 × 7**	7.04	1.96	−12.22	5.11	−11.97	−30.95	8.96	21.41	1.59	8.22	−1.38	−24.38	7.73	7.81	−15.24
**4 × 8**	10.25	31.16	27.81	5.19	−22.13	−29.86	10.96	33.66	24.26	10.03	9.50	−7.73	10.92	43.97	19.74
**5 × 6**	7.46	14.15	12.18	6.04	−2.03	−15.99	10.60	11.70	7.29	9.56	2.91	−11.40	9.45	14.06	1.94
**5 × 7**	7.25	18.76	12.93	4.24	−9.30	−17.51	7.37	−1.99	−19.01	7.86	15.67	0.90	7.46	19.17	2.19
**5 × 8**	8.27	17.89	8.67	4.86	−12.20	−18.04	9.80	17.51	7.69	9.10	19.42	16.82	8.18	22.55	12.05
**6 × 7**	6.77	8.84	1.80	4.90	−14.04	−31.85	8.65	9.36	−12.45	8.26	−0.42	−23.45	7.64	5.45	−17.58
**6 × 8**	8.81	23.56	15.77	5.05	−23.02	−14.84	11.01	26.12	11.44	9.19	0.77	−14.83	10.64	38.90	14.78
**7 × 8**	8.55	27.61	12.35	5.02	−0.99	−15.35	9.68	43.20	27.70	8.21	23.92	10.20	9.57	69.83	58.18
**S.E.**	0.762	0.726	0.838	0.476	0.583	0.674	0.433	0.531	0.613	0.366	0.449	0.518	0.452	0.553	0.639

^†^ The names of the parents are: 1 = Vedrantais; 2 = Ita1; 3 = Ogen; 4 = Top Mark; 5 = Magyar Kincs; 6 = Hale’s Best Jumbo; 7 = PI414723; 8 = PI161375.

**Table 8:** LsM, SCA and its ranking (in italics), MPH, and BPH for earliness (DTR) of the 28 crosses in environments with a significant ANOVA SCA mean square.

	Latina 2014					Perugia 2014					Latina 2015					Latina 2016					Perugia 2016				
Cross ^†^	LsM	SCA	*Rank*	MPH	BPH	LsM	SCA	*Rank*	MPH	BPH	LsM	SCA	*Rank*	MPH	BPH	LsM	SCA	*Rank*	MPH	BPH	LsM	SCA	*Rank*	MPH	BPH
**1 × 2**	64.1	−0.107	*12*	−0.77	1.91	68.1	−2.771	*1*	−9.56	−9.20	64.7	−0.678	*8*	−1.07	3.52	66.3	−0.610	*6*	−0.90	1.53	66.7	−4.216	*2*	−10.77	−6.97
**1 × 3**	62.5	0.132	*17*	−0.56	−0.48	69.9	0.229	*17*	−4.57	−2.24	62.7	−0.467	*12*	1.13	1.95	65.5	0.157	*17*	−2.46	0.31	71.9	0.329	*14*	−1.44	5.58
**1 × 4**	64.4	1.587	*27*	0.95	2.38	73.1	1.462	*22*	−3.12	−2.53	63.7	0.922	*22*	0.24	1.92	67.4	2.335	*28*	1.53	3.22	76.7	1.695	*24*	−1.68	−1.41
**1 × 5**	62.1	−0.057	*14*	−0.64	0.00	69.7	0.917	*20*	−2.79	1.90	62.3	−0.356	*13*	0.32	0.97	63	−0.843	*4*	−1.79	0.00	68.1	−0.749	*8*	−6.71	−0.15
**1 × 6**	62.3	0.921	*25*	−0.80	−0.64	71.4	1.906	*25*	−2.26	0.42	63.1	0.433	*18*	−0.47	0.96	63.9	−0.387	*10*	−2.07	−1.99	74.5	2.162	*25*	−2.04	0.27
**1 × 7**	58.5	−0.063	*13*	−1.60	4.46	64.5	−0.205	*16*	−11.64	−9.15	57.7	1.022	*25*	−3.19	1.76	60.3	1.502	*26*	−3.05	2.03	66.7	0.440	*18*	−11.13	−7.75
**1 × 8**	58.7	−2.413	*1*	−7.27	−6.68	67.8	−1.538	*5*	−5.83	−1.74	60.1	−0.878	*6*	−1.31	1.35	62	−2.154	*2*	−2.82	−0.48	72.2	0.340	*15*	−3.35	0.84
**2 × 3**	65.4	−0.529	*5*	1.32	4.14	68.7	−2.038	*2*	−6.59	−3.92	67.5	0.522	*20*	4.01	9.76	68.1	−0.421	*9*	−0.95	−0.58	66.6	−2.716	*3*	−4.72	−2.20
**2 × 4**	66.9	0.593	*21*	2.14	3.40	74.6	1.929	*26*	−1.39	−1.32	65.3	−1.356	*3*	−1.73	1.08	67.7	−0.510	*7*	−0.44	0.30	76.5	3.784	*26*	2.14	6.69
**2 × 5**	66.2	0.482	*20*	3.12	6.60	69.3	−0.616	*12*	−3.75	1.32	68.4	1.900	*27*	5.23	10.86	67.8	0.846	*25*	3.12	7.62	66.7	0.073	*13*	−4.65	−2.20
**2 × 6**	62.7	−2.274	*2*	−2.79	0.00	70.2	−0.360	*14*	-4.29	-1.27	65	−1.444	*2*	−1.96	1.09	67.1	−0.298	*11*	0.37	2.91	69.1	−1.083	*7*	−5.34	−3.63
**2 × 7**	62.3	0.176	*18*	1.88	11.25	65.4	−0.338	*15*	−10.78	−7.89	58.6	−1.922	*1*	−6.24	3.35	60.7	−1.276	*3*	−4.86	2.71	61.5	−2.538	*4*	−14.58	−14.23
**2 × 8**	66.3	1.660	*28*	2.00	4.08	74.6	4.195	*28*	3.18	8.12	67.8	2.978	*28*	6.27	14.33	69.5	2.268	*27*	6.27	11.56	76.3	6.695	*28*	6.49	6.56
**3 × 4**	64.3	−0.302	*9*	0.86	2.39	72	0.529	*19*	−2.17	0.70	65.4	0.922	*23*	3.73	6.34	67	0.324	*19*	−1.83	−0.74	74.1	0.729	*21*	1.37	8.81
**3 × 5**	64.7	0.721	*23*	3.60	4.19	70.4	1.717	*24*	0.64	2.92	64.9	0.511	*19*	5.36	5.53	65.6	0.146	*16*	−0.61	4.13	65.1	−2.116	*6*	−4.48	−4.41
**3 × 6**	62.8	−0.368	*8*	0.08	0.16	68.5	−0.894	*10*	−3.93	−3.66	63.5	−0.833	*7*	0.95	3.25	66.3	0.335	*20*	−1.19	1.69	70.1	−0.671	*9*	−1.54	2.94
**3 × 7**	60.1	−0.285	*10*	1.18	7.32	66.1	1.595	*23*	−7.23	−6.90	58.2	−0.178	*14*	−1.52	2.65	59.7	−0.776	*5*	−6.79	1.02	65.3	0.606	*19*	−6.98	−4.11
**3 × 8**	63.5	0.632	*22*	0.40	1.11	68.1	−1.138	*8*	−3.06	−1.30	62.2	−0.478	*11*	2.98	4.89	66	0.235	*18*	0.53	5.94	74.1	3.840	*27*	6.08	8.81
**4 × 5**	63.4	−0.957	*3*	0.00	2.09	69.4	−1.183	*7*	−3.68	1.46	62.7	−1.300	*4*	−0.71	1.62	65	−0.143	*13*	−0.38	3.17	71.3	0.651	*20*	−2.53	4.55
**4 × 6**	63.3	−0.246	*11*	−0.63	0.96	72.3	1.073	*21*	−1.50	1.69	65	1.089	*26*	0.85	1.09	65.7	0.113	*15*	−0.98	0.77	74.6	0.429	*16*	−2.10	0.40
**4 × 7**	60.9	0.104	*16*	0.91	8.75	64.4	−2.038	*3*	−12.20	−9.30	58.9	0.944	*24*	−2.89	3.88	60.5	0.335	*21*	−4.42	2.37	68.1	−0.027	*12*	−9.44	−5.81
**4 × 8**	62.5	−0.779	*4*	−2.65	−1.88	69.3	−1.771	*4*	−4.22	0.43	61.1	−1.222	*5*	−1.37	3.04	63	−2.454	*1*	−2.93	1.12	66.4	−7.260	*1*	−11.29	−7.26
**5 × 6**	62.9	−0.024	*15*	0.80	1.29	69	0.529	*18*	−1.08	0.88	64.3	0.544	*21*	2.06	4.21	64.1	−0.265	*12*	0.00	1.75	69.3	1.251	*22*	−2.74	1.61
**5 × 7**	59.7	−0.407	*7*	1.10	6.61	63.1	−0.516	*13*	−9.47	−7.75	57.2	−0.667	*9*	−3.38	0.88	58.4	−0.510	*8*	−4.34	−1.18	63.5	1.529	*23*	−9.61	−6.89
**5 × 8**	62.9	0.243	*19*	0.00	1.29	67.5	−0.849	*11*	−1.75	−1.32	61.5	−0.633	*10*	1.65	3.71	65	0.768	*23*	3.75	4.33	66.9	−0.638	*10*	−4.29	−1.91
**6 × 7**	60.3	0.904	*24*	1.60	7.68	63.4	−0.927	*9*	−10.77	−10.70	58.2	0.389	*16*	−3.80	2.65	59.3	−0.054	*14*	−4.59	0.34	65.9	0.440	*17*	−10.10	−8.85
**6 × 8**	63.0	1.087	*26*	−0.32	0.48	67.7	−1.327	*6*	−3.35	−1.88	61.9	−0.178	*15*	0.16	4.38	65.3	0.557	*22*	2.43	4.82	68.5	−2.527	*5*	−6.10	−4.33
**7 × 8**	58.7	−0.429	*6*	−1.92	4.82	66.6	2.429	*27*	−4.86	−3.48	56.6	0.411	*17*	−2.41	−0.18	60	0.779	*24*	−1.15	1.52	64.5	−0.449	*11*	−10.35	−9.92
**S.E.**	0.229	0.529		0.765	0.883	0.551	1.151		1.667	1.925	0.311	0.561		0.812	0.938	0.270	0.755		1.093	1.262	0.741	1.585		2.297	2.652

^†^ The names of the parents are: 1 = Vedrantais; 2 = Ita1; 3 = Ogen; 4 = Top Mark; 5 = Magyar Kincs; 6 = Hale’s Best Jumbo; 7 = PI414723; 8 = PI161375.

## Supplementary Materials

The following are available online at https://www.mdpi.com/2223-7747/9/2/282/s1, Figure S1: Representative fruits of the used inbred lines; Table S1: Orthogonal comparisons between hybrids and parental lines; Table S2: Combined ANOVA for fruit weight (Kg), length (cm), diameter (cm) and shape (length/diameter) across the six environments used in the experiment; Table S3: Genetic Distance matrix among parental lines.
