# Supplementary material for: Exploring Heterosis in Melon (Cucumis melo L.)
Source: Plants (Basel). 2020 Feb 21;9(2):282. doi: 10.3390/plants9020282 (PMC7076541; doi:10.3390/plants9020282)
Supplement: Supplementary file 1 [file plants-09-00282-s001.zip › supplementary/Figure S1.pdf]

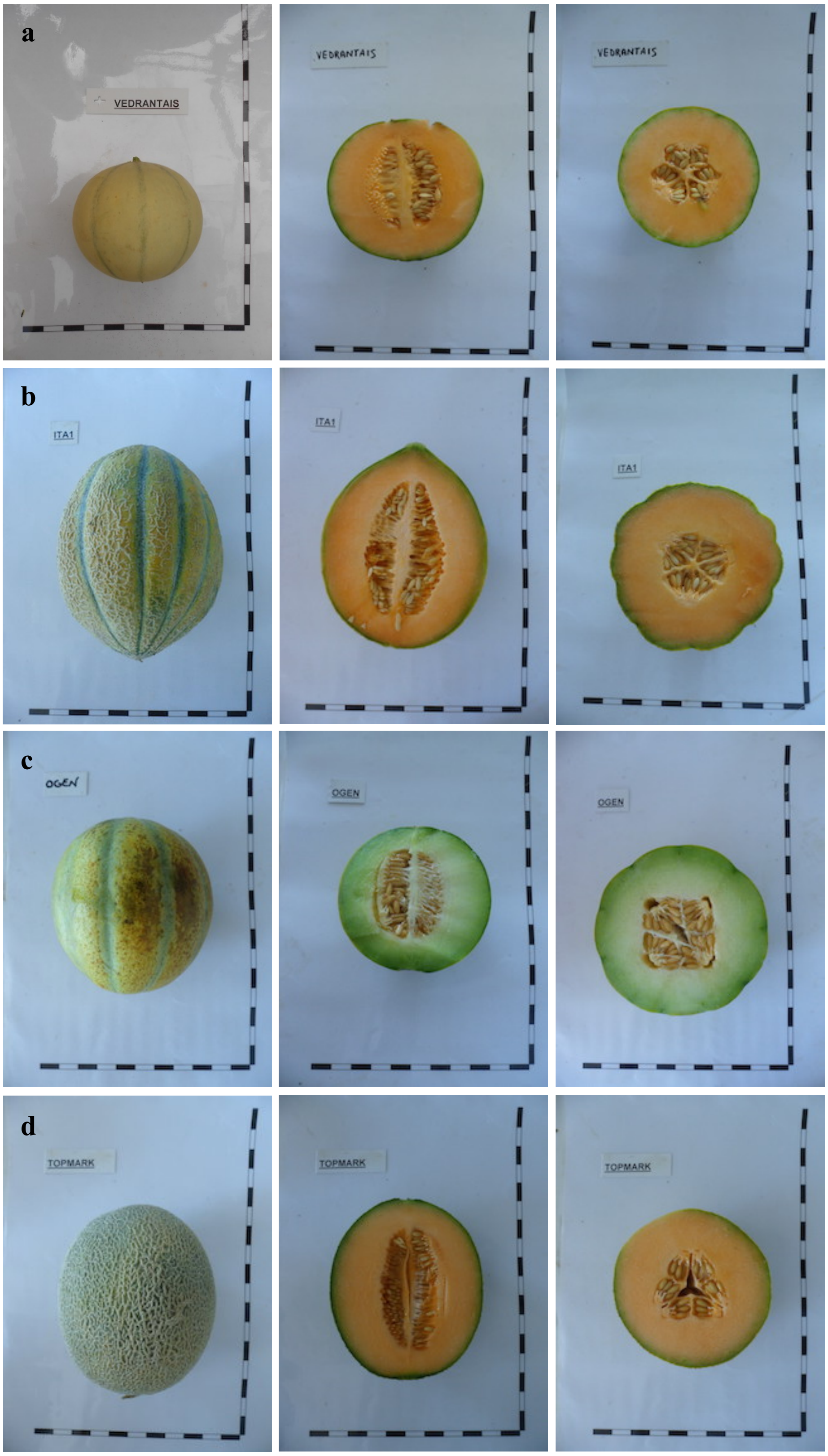
a
VEDRANTAIS
VEDRANTAIS
VEDRANTAIS
b
ITA1
ITA1
ITA1
c
OGEN
OGEN
OGEN
d
TOPMARK
TOPMARK
TOPMARK

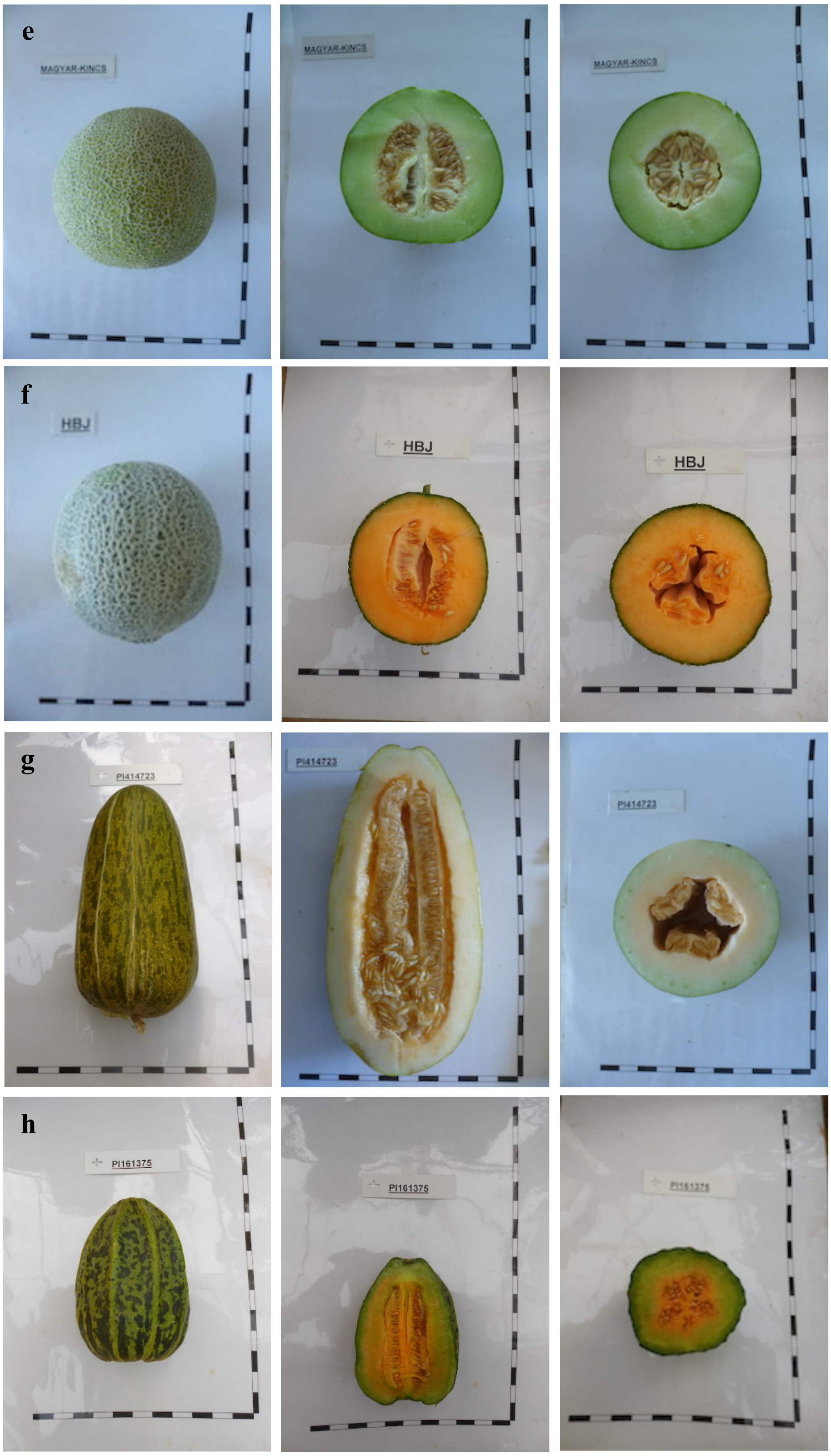

Figure S1. Representative fruits of the used inbred lines: (a) VEDRANTAIS (cantalupensis); (b) ITA1 (reticulatus); (c) OGEN (cantalupensis); (d) TOP MARK (reticulatus); (e) MAGYAR KINCS (reticulatus); (f) HALE'S BEST JUMBO (reticulatus); (g) PI414723 (momordica); (h) PI161375 (chinensis); bar 2 cm.
