# Supplementary material for: Exploring Heterosis in Melon (Cucumis melo L.)
Source: Plants (Basel). 2020 Feb 21;9(2):282. doi: 10.3390/plants9020282 (PMC7076541; doi:10.3390/plants9020282)
Supplement: Supplementary file 1 [file plants-09-00282-s001.zip › supplementary/Table S1.doc]

**Table S1**. Orthogonal comparisons between hybrids and parental lines for yield per plant, total soluble solids (TSS) and days to ripening (DTR) in each of the six environments.

|  |  | **Estimate** | **SE** | **df** | **t.ratio** |  |
| --- | --- | --- | --- | --- | --- | --- |
| Yield | Latina 2014 | 2.94 | 0.367 | 72 | 8.0 | *** |
|  | Perugia 2014 | 1.84 | 0.192 | 72 | 9.6 | *** |
|  | Latina 2015 | 1.94 | 0.45 | 72 | 4.3 | *** |
|  | Perugia 2015 | 1.90 | 0.308 | 72 | 6.2 | *** |
|  | Latina 2016 | 3.94 | 0.507 | 72 | 7.8 | *** |
|  | Perugia 2016 | 4.15 | 0.383 | 72 | 10.8 | *** |
| TSS | Latina 2014 | 1.02 | 0.079 | 3520 | 12.9 | *** |
|  | Perugia 2014 | -0.18 | 0.109 | 1774 | -1.6 | ns |
|  | Latina 2015 | 1.31 | 0.077 | 3161 | 17.0 | *** |
|  | Perugia 2015 | 0.85 | 0.097 | 2186 | 8.8 | *** |
|  | Latina 2016 | 1.57 | 0.099 | 3661 | 15.9 | *** |
|  | Perugia 2016^‡^ | NA | NA | NA | NA |  |
| DTR | Latina 2014 | 0.06 | 0.138 | 499 | 0.4 | ns |
|  | Perugia 2014 | -3.42 | 0.255 | 504 | -13.4 | *** |
|  | Latina 2015 | 0.07 | 0.142 | 504 | 0.5 | ns |
|  | Perugia 2015 | -2.81 | 0.278 | 504 | -10.1 | *** |
|  | Latina 2016 | -0.35 | 0.171 | 500 | -2.0 | * |
|  | Perugia 2016 | -3.43 | 0.331 | 504 | -10.3 | *** |

*, **, *** Significant at the 0.05, 0.01 and 0.001 probability level, respectively; ns, not significant

^‡^ NA, data not available
