# Supplementary material for: Exploring Heterosis in Melon (Cucumis melo L.)
Source: Plants (Basel). 2020 Feb 21;9(2):282. doi: 10.3390/plants9020282 (PMC7076541; doi:10.3390/plants9020282)
Supplement: Supplementary file 1 [file plants-09-00282-s001.zip › supplementary/Table S2.doc]

| **Table S2.** Combined Anova for fruit weight (Kg), length (cm), diameter (cm) and shape (length/diameter) across the six environments used in the experiment. | | | | | | | | |  |
| --- | --- | --- | --- | --- | --- | --- | --- | --- | --- |
|  |  | **Weight** |  | **Length** |  | **Diameter** |  | **Shape** | |
|  | df | MS |  | MS |  | MS |  | MS | |
| Environments | 5 | 85.20*** |  | 2169.18*** |  | 605.22*** |  | 8.670*** | |
| Entries | 27 | 53.38*** |  | 9045.75*** |  | 410.76*** |  | 71.50*** | |
| GCA | 7 | 5.28*** |  | 1198.29*** |  | 40.78*** |  | 9.81*** | |
| SCA | 20 | 0.94*** |  | 54.50*** |  | 4.81*** |  | 0.19*** | |
| Env × Entries | 135 | 1.07*** |  | 24.86*** |  | 13.09*** |  | 0.18*** | |
| Env × GCA | 35 | 0.04 |  | 3.06 |  | 0.80 |  | 0.04*** | |
| Env × SCA | 100 | 0.04 |  | 0.86 |  | 0.28 |  | 0.00 | |
| Error | 13257 | 0.15 |  | 4.29 |  | 1.66 |  | 0.02 | |
|  |  |  |  |  |  |  |  |  | |
| GCA Variance (σ^2^_g_) |  | 0.0401*** |  | 10.5703*** |  | 0.3282*** |  | 0.0887*** | |
| SCA Variance (σ^2^_s_) |  | 0.0502*** |  | 2.9799*** |  | 0.2517*** |  | 0.0104*** | |
| GCA × Env Variance |  | 0.0002*** |  | 0.1220*** |  | 0.0287*** |  | 0.0022*** | |
| SCA × Env Variance |  | 0*** |  | 0*** |  | 0*** |  | 0*** | |
| Error Variance (σ^2^_E_) |  | 0.1506 |  | 4.2875 |  | 1.6578 |  | 0.0183 | |
| Additive Variance (σ^2^_A_) |  | 0.0802 |  | 21.1406 |  | 0.6565 |  | 0.1774 | |
| Dominance Variance (σ^2^_D_) |  | 0.0502 |  | 2.9799 |  | 0.2517 |  | 0.0104 | |
| Phenotypic Variance (σ^2^_P_) |  | 0.2810 |  | 28.4081 |  | 2.5659 |  | 0.2061 | |
| GCA-SCA ratio (σ^2^_g_/σ^2^_s_) |  | 0.80 |  | 3.55 |  | 1.30 |  | 8.55 | |
| Narrow sense Heritability (h^2^_N_) |  | 0.29 |  | 0.74 |  | 0.26 |  | 0.86 | |
| Broad sense Heritability (h^2^_B_) |  | 0.46 |  | 0.85 |  | 0.35 |  | 0.91 | |

***Significant at the 0.001 probability level.
