# Supplementary material for: Exploring Heterosis in Melon (Cucumis melo L.)
Source: Plants (Basel). 2020 Feb 21;9(2):282. doi: 10.3390/plants9020282 (PMC7076541; doi:10.3390/plants9020282)
Supplement: Supplementary file 1 [file plants-09-00282-s001.zip › supplementary/Table S3.doc]

**Table S3.** Genetic Distance matrix among parental lines

|  | **PI161375** | **PI414723** | **HALE'S BEST JUMBO** | **MAGYAR KINCS** | **TOP MARK** | **OGEN** | **ITA1** | **VEDRANTAIS** |
| --- | --- | --- | --- | --- | --- | --- | --- | --- |
| **PI161375** | 0 | 0.344 | 0.518 | 0.550 | 0.538 | 0.548 | 0.507 | 0.499 |
| **PI414723** | 0.344 | 0 | 0.436 | 0.439 | 0.464 | 0.436 | 0.440 | 0.430 |
| **HALE'S BEST JUMBO** | 0.518 | 0.436 | 0 | 0.352 | 0.142 | 0.344 | 0.253 | 0.264 |
| **MAGYAR KINCS** | 0.550 | 0.439 | 0.352 | 0 | 0.355 | 0.266 | 0.350 | 0.341 |
| **TOP MARK** | 0.538 | 0.464 | 0.142 | 0.355 | 0 | 0.355 | 0.250 | 0.286 |
| **OGEN** | 0.548 | 0.436 | 0.344 | 0.266 | 0.355 | 0 | 0.330 | 0.336 |
| **ITA1** | 0.507 | 0.440 | 0.253 | 0.350 | 0.250 | 0.330 | 0 | 0.247 |
| **VEDRANTAIS** | 0.499 | 0.430 | 0.264 | 0.341 | 0.286 | 0.336 | 0.247 | 0 |
